# ﻿Recommendations on approving the name “ *Entomosporium*”, with a new species, *E.dichotomanthes* from China (Leotiomycetes, Drepanopezizaceae)

**DOI:** 10.3897/mycokeys.107.121962

**Published:** 2024-07-10

**Authors:** Hong De Yang, Ruvishika S. Jayawardena, Xiang Yu Zeng, Vinodhini Thiyagaraja, Qi Zhao, Kevin D. Hyde

**Affiliations:** 1 Key Laboratory of Phytochemistry and Natural Medicines, Kunming Institute of Botany, Chinese Academy of Sciences, Kunming 650201, China; 2 Center of Excellence in Fungal Research, Mae Fah Luang University, Chiang Rai 57100, Thailand; 3 School of Science, Mae Fah Luang University, Chiang Rai 57100, Thailand; 4 Department of Plant Pathology, College of Agriculture, Guizhou University, Guiyang 550025, China

**Keywords:** *Diplocarpon*, *Hymenulacerealis*, plant pathogen, phylogeny, *Pseudopezizamedicaginis*

## Abstract

The phytopathogenic genus, *Entomosporium* can cause serious leaf diseases worldwide. *Entomosporium* has long been regarded as a synonym of *Diplocarpon*. However, different morphologies between *Entomosporium* and *Diplocarpon* make this doubtful. Based on morpho-phylogenetic analyses, the placement of the genus was re-evaluated in this study. The combined the internal transcribed spacer gene region (ITS) and the 28S large subunit ribosomal RNA gene region (LSU) phylogenetic analysis shows that *Entomosporium* is an independent clade within Drepanopezizaceae and formed a sister clade to the generic type *Diplocarpon*. Moreover, *Hymenula* and *Pseudopeziza* do not cluster in Drepanopezizaceae. We propose to resurrect the name *Entomosporium*, and exclude *Hymenulacerealis* and *Pseudopezizamedicaginis* from Drepanopezizaceae and propose to treat them under Ploettnerulaceae. A new species, *E.dichotomanthes* is also introduced from China based on morpho-molecular analyses which is associated with *Dichotomanthestristaniicarpa*.

## ﻿Introduction

*Entomosporium* Lév, a synonym of *Diplocarpon* F.A. Wolf, is a member of the strongly plant-pathogenic family Drepanopezizaceae (Holtslag et al. 2003; Nunes et al. 2016; Johnston et al. 2019; Wöhner and Emeriewen 2019). The *Entomosporium* species causes entomosporium leaf disease worldwide and frequently occurs as an epidemic (Bogo et al. 2018). As many species are described without molecular data, the relationship with *Diplocarpon* species remains unclear. Although *Diplocarpon* species are common and widespread, studies on *Diplocarpon* have predominantly focused on their phytopathology, with the taxonomy utilizing molecular markers being largely overlooked (Wijayawardene et al. 2017; Ekanayaka et al. 2019).

Historically, *Diplocarpon* has undergone several revisions. *Diplocarpon* was erected by Wolf (1912), with the type species *D.rosae* (syn. *Asteromarosae*) which caused black spot disease on Rose. The sexual stage of *Diplocarpon* forms cup-like apothecia, in which the asci develop. The ascospores are hyaline, 2-celled, marssonina-like and oblong-elliptical (Wolf 1912; Stowell and Backus 1967). The asexual stage comprises acervuli that develop on leaf surfaces accompanied by typical black dot disease (Frick 1943). Conidia of *D.rosae* are hyaline, oblong-elliptical, 2-celled with one constricted septum. By the interpretations of the morphology of these taxa, a broad concept of species circumscription was employed. Some members of *Bostrichonema*, *Entomosporium*, *Gloeosporium* and *Marssonina* were treated as synonyms of *Diplocarpon*. For example, *Ascochytacoronariae* (= *D.coronariae*), *Bostrichonemaalpestre* (= *D.alpestre*), *Dothideaimpressa* (= *D.impressa*), *Leptothyriumfragariae* (= *D.fragariae*) and *Phacidiumsaponariae* (= *D.saponariae*) were transferred into *Diplocarpon* (Johnston et al. 2014; Braun 2018; Crous et al. 2020). In addition, all members in *Entomopeziza*, *Entomosporium* and *Morthiera* were regarded as congruent with *D.mespili* (Stowell and Backus 1966; Gamundí et al. 2004; Johnston et al. 2014).

Although genera, such as *Entomopeziza*, *Entomosporium* and *Morthiera* have morphological similarities to *Diplocarpon*, it is perplexing that they are considered as synonyms. For example, 15 epithets of *Entomosporium* were regarded as *D.mespili*, as the sexual stage of *Entomosporium* morphologically resembles *Diplocarpon* (Naoui 2013; Johnston et al. 2014). However, *Entomosporium* produces cruciform, insect-like, 2–6-celled conidia, which is distinct from the conidia of *Diplocarpon* (Stowell and Backus 1966). Moreover, *Entomosporium* species are widely distributed in Argentina, Australia, Brazil, Canada, China, India, Israel, Italy, Japan, New Zealand, North America, Pakistan, and South Africa, on a wide host range of Rosaceae (Stowell and Backus 1966; Cariddi et al. 2009; Batool et al. 2014). *Diplocarpon* on the other hand, is mostly or specifically parasitic on herbaceous Rosaceae or low shrubs. The proposal to adopt *Diplocarpon* over *Entomosporium* is doubtful (Horie and Kobayashi 1980; Wijayawardene et al. 2021). The hypothesis that *Diplocarponmespili* did not speciate with its worldwide spread should be re-evaluated (Chethana et al. 2021). An example of evidence is that *Entomosporium* sp. from Japan has more lateral cells (2–4) (Horie and Kobayashi 1979). Chen et al. (2022) introduced a new species with insect-like conidia but under the name “*Diplocarpon*”. In Index Fungorum (https://www.indexfungorum.org, 23 Nov 2023), 12 *Diplocarpon* species are recorded, namely *D.alpestre*, *D.coronariae*, *D.earlianum*, *D.fragariae*, *D.hymenaeae*, *D.impressum*, *D.mali*, *D.mespili*, *D.mespilicola*, *D.polygoni*, *D.rosae* and *D.saponariae*.

We are studying the pathogens of urban and forest tree species in Yunnan Province (Thiyagaraja et al. 2024) and in this study *Entomosporium* leaf disease was found in *Dichotomanthestristaniicarpa* and has not been reported before. *Dichotomanthes* is endemic to Yunnan and Sichuan provinces in China (Zhou et al. 2000). It belongs to Rosaceae, with only one species *D.tristaniicarpa* which is a rare evergreen shrub tree, and is used as ornamental and medicinal plants (Tang et al. 2010; Yang et al. 2018). The ITS sequence blastn search of the newly generated sequences showed the close hits to *Diplocarpon*, and identified it as a new species based on the evidence from both morphology and phylogeny. Since the increasing number of members and updating molecular data of *Diplocarpon*, this study has provided an opportunity for a better understanding of the taxonomy of the genus. In this study, we interpret the relationship between *Entomosporium* and *Diplocarpon*, and further re-evaluate the taxonomy of Drepanopezizaceae.

## ﻿Materials and methods

### ﻿Sampling, isolation and morphological observations

Leaves with lesions of *Dichotomanthestristaniicarpa* were collected from Yunnan Province. For single-spore isolation, the fruit bodies were transferred to sterilized water in a centrifuge tube using a syringe needle, then crushed into pieces using pipette tips. Subsequently, 200 μL of the spore suspension was transferred to potato dextrose agar (PDA) using a micropipette (Zhang et al. 2013). For tissue isolation, the leaves were washed with distilled water for 1 minute and then air-dried. The margins of the disease lesions were cut into fragments (0.5 × 0.5 cm) under aseptic conditions. These fragments were surface-sterilized with 75% ethanol for 30 seconds, followed by dipping in 1% sodium hypochlorite for 40 seconds. They were then rinsed three times in sterile demineralized distilled water before being transferred onto a PDA plate, with four fragments per plate (Senanayake et al. 2020). The Petri dishes were incubated in the dark at 25 °C. Specimens were deposited at the Herbarium of Kunming Institute of Botany, Chinese Academy of Sciences (KUN-HKAS). Morphological observations were performed using Nikon SMZ745T dissecting microscope (DM) and Nikon Eclipse 80i compound microscope, equipped with IMG Camera SC2000C. Index Fungorum and Facesoffungi numbers were obtained as in Index Fungorum (https://www.indexfungorum.org/) and Jayasiri et al. (2015) and the details of the fungus were deposited in the Greater Mekong Subregion database (Chaiwan et al. 2021).

### ﻿DNA extraction, PCR amplification and sequencing

Genomic DNA was extracted by using Lysis Buffer for Microorganism to Direct PCR (Takara), following the user manual. PCR amplifications were performed in T100 Thermal Cycler (T100™, Bio-Rad, USA) with ingredients of 21 µL GoldenStar T6 Super PCR Mix (Tsingke), 1 µL (10 µM) of each primer and 2 µL DNA template. Amplification conditions include 3 min initial denaturation at 95 °C, followed by 35 cycles of 95 °C denaturation for 15 s, 53 °C ~ 56 °C annealing for 15 min, 72 °C extension for 20 s, followed by a final extension at 72 °C for 5 min. The primer set ITS5/ITS4 (White et al. 1990) was used to amplify the internal transcribed spacer gene region (ITS); and LROR/LR5 for the 28S large subunit ribosomal RNA gene region (LSU) (Vilgalys and Hester 1990; White et al. 1990) and 983F/2218R for translation elongation factor 1-alpha gene region (*tef*-α) (Rehner and Buckley 2005). PCR products were purified and sequenced by Sangon Biotech (Shanghai) Co., Ltd., Shanghai, China.

### ﻿Phylogenetic analyses

Reverse and forward sequences were assembled using Chromas Pro (2.1.8) and initial identification was subjected to the NCBI (https://www.ncbi.nlm.nih.gov/) using BLAST search. Sequences of similar taxa were retrieved from the NCBI, and additional reference sequence selections based on Johnston et al. (2019) were downloaded from the DataStore (https://datastore.landcareresearch.co.nz/). The alignment was constructed with the online tool MAFFT v.7 (http://mafft.cbrc.jp/alignment/server) (Katoh and Standley 2013), and refined using BioEdit v. 7.7.1 (Hall 1999). The final combined data matrix was converted by the online tool ALTER (https://www.sing-group.org/ALTER/) (Glez-Peña et al. 2010). A quick Phylogenetic analysis was conducted using OFPT (Zeng et al. 2023) following its default protocol. The final Phylogenetic analyses were conducted on the CIPRES Science Gateway platform (https://www.phylo.org), using tools of RAxML-HPC v.8 on XSEDE (8.2.12) for maximum likelihood (ML) and MrBayes on XSEDE (3.2.7a) for Bayesian inference (BI). In the Bayesian inference, the best optimal substitution model was determined by using ModelFinder (Kalyaanamoorthy et al. 2017) under the Bayesian information criterion (BIC). The final phylogenetic tree was visualized with FigTree v. 1.4.4 and edited using Adobe Photoshop CS6 version 10.0. Sequences of the new strain generated in this study are deposited in GenBank (Table 1).

**Table 1.** GenBank accession numbers used in the phylogenetic analyses.

Species	Strain	Country	GenBank accession number	
			ITS	LSU
*Acephalaapplanata*	CBS 109321T	Switzerland	NR_119482	KF951051
*Blumeriellahiemalis*	CBS 146.35	USA	MH855609	MH867119
*B.kerriae*	JS20160615	United Kingdom	KY929501	–
*Cadophorafascicularis*	CBS 146382	Germany	NR_170729	MN339414
*Cheirosporabotryospora*	MFLUCC 17-1399	China	MN535816	MN535856
*Collembolisporaaristata*	CPC 21145T	Czech Republic	NR_111830	NG_042760
*Cylindrosporiumconcentricum*	CBS:157.35	Australia	MH855615	MH867125
*Diplocarponcoronariae*	Satoko Kanematsu Dip-ap6-3	Japan	AB609188	–
*D.coronariae*	CS 01	Korea	AB494960	AB494964
*D.coronariae*	5C11	USA	MW364818	–
*D.coronariae*	NL1	China	KY672995	–
*D.earlianum*	CBS 162.32	Unknown	MH855259	MH866712
*D.rosae*	CBS 163.31	Unknown	MH855164	MH866612
*D.rosae*	CBS 829.72	Netherlands	–	MH872311
*D.rosae*	CFCC6814	Unknown	KP099199	–
*D.rosae*	DortE4^T^	Germany	Genome	Genome
*Drepanopezizabalsamiferae*	14-19	USA	MN315242	–
*D.brunnea*	Marbr1	Unknown	genome	genome
*D.ribis*	CBS:200.36	Netherlands	MH855774	MH867284
*D.salicis*	CBS:405.64	Switzerland	MH858467	MH870102
*D.tremulae*	CBS 408.64	Switzerland	MH858468	MH870103
*D.triandrae*	CBS 409.64	Switzerland	MH858469	MH870104
***Entomosporiumdichotomanthes***	**HKAS 131154**	**China**	**PP333041**	**PP333042**
*E.mespili*	CBS 166.28	England	–	MH877689
*E.mespili*	CBS 402.65	Unknown	–	MH870277
*E.mespili*	KACC 42361	Korea	EF600984	–
*E.mespili*	KACC 42436	Korea	EF600985	–
*E.mespilicola*	CF 2	China	OM237437	MW809414
*E.mespilicola*	CF 3	China	OM237438	MW809415
*E.mespilicola*	CGMCC3 20492^T^	China	OM237436	MW809413
*Hyaloscyphaericae*	UAMH 6735^T^	Canada	AF284122	MH018947
*H.gabretae*	CBS 145341^T^	Czech Republic	MZ520780	NG_081311
*H.gryndleri*	CBS 145337^T^	Czech Republic	MZ520785	MZ520774
*H.minuta*	G.M.2015-04-06.2^T^	Luxembourg	KY769526	–
*H.vitreola*	Huhtinen M220^T^	Finland	FJ477059	FJ477058
*Hymenulacerealis*	CBS 132.34^T^	Japan	NR_171209	NG_070839
*H.cerealis*	CBS 540.63	United Kingdom	MH858350	MH869971
*Leuconeurosporacapsici*	CBS:176.44	Netherlands	MH856125	MH867637
*L.pulcherrima*	AFTOL-ID 1397	Unknown	KF049206	FJ176884
*Meliniomycesvariabilis*	UAMH 8861^T^	Canada	NR_121313	NG_073616
*Mycochaetophoragentianae*	MAFF 239231^T^	Japan	NR_121201	AB496937
*Neospermosporaavenae*	CBS 227.38^T^	USA	MW298276	NG_077377
*Oculimaculayallundae*	CBS 110665^T^	South Africa	MW810278	MW715035
*Pseudaegeritacorticalis*	ICMP 15324^T^	New Zealand	EF029224	–
*Pseudopezizamedicaginis*	CBS 283.55	USA	MH857484	MH869025
*Rhexocercosporidiumcarotae*	CBS 418.65^T^	Norway	NR_111086	MH870289
*Rhynchosporiumagropyri*	CBS:146762	Switzerland	MW298346	MW298448
*Thedgonialigustrina*	CBS 132025	Korea	GU269839	GU253856
*T.ligustrina*	CBS:148.59^T^	Netherlands	NR_175086	NG_078647
*T.ligustrina*	CPC 10530	Netherlands	FJ839628	FJ839665
*Vibrisseatruncorum*	AFTOL-ID 1322^T^	Canada	EU434854	FJ176874
*Ypsilinabuttingtonensis*	CPC 39109^T^	United Kingdom	NR_170831	MT373355

Type strains are marked with “T”, and strains from the present study are in black bold.

## ﻿Results

**Figure 1.**
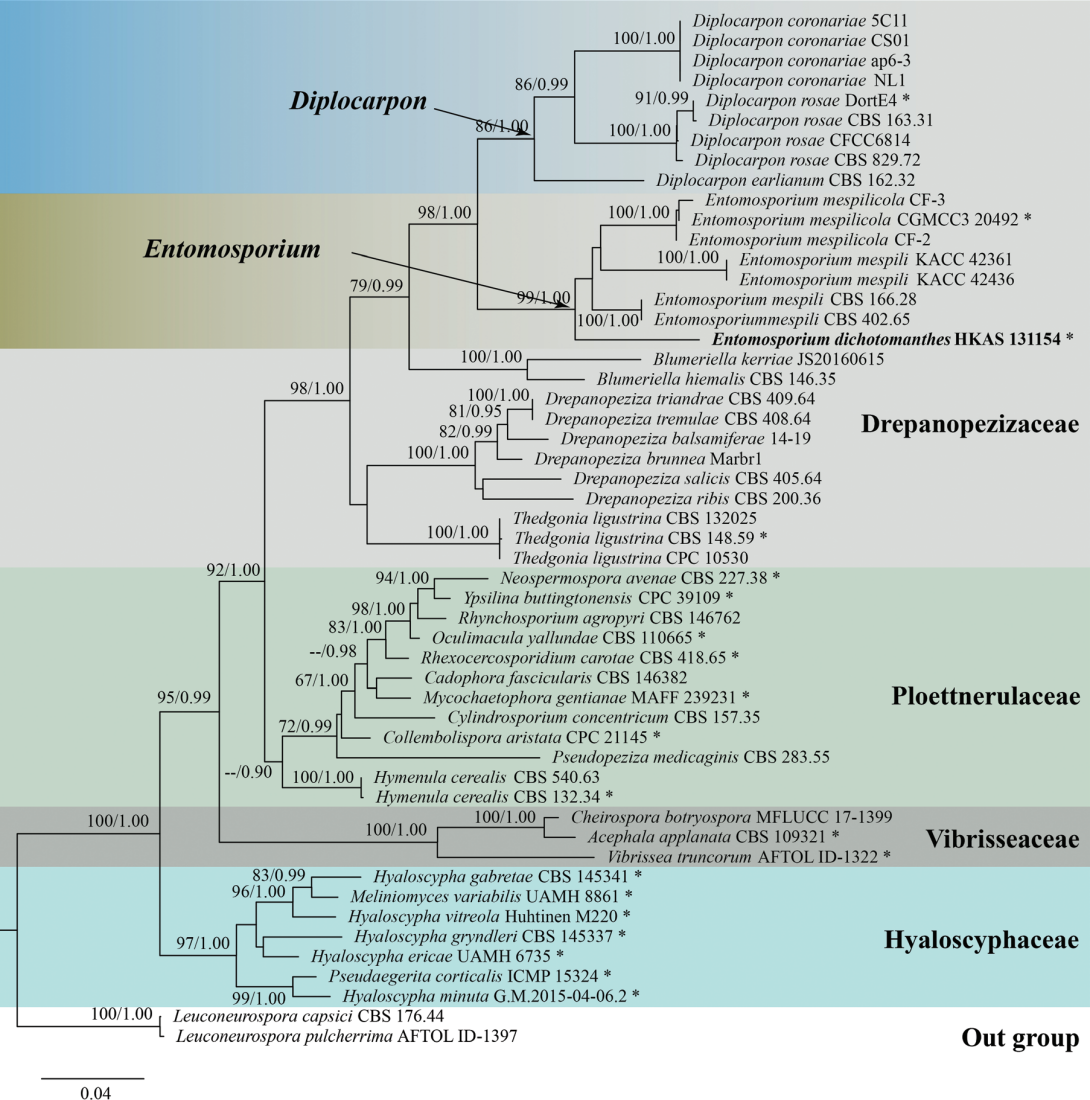
Maximum likelihood phylogenetic tree inferred from combined LSU and ITS sequence data of Drepanopezizaceae and its closely related families. The tree is artificially rooted with *Leuconeurosporacapsici* (CBS:176.44) and *Leuconeurosporapulcherrima* (AFTOL-ID 1397). Maximum likelihood bootstrap values ≥65% and Bayesian Posterior Probabilities (BYPP) ≥ 0.90 are given at the nodes. Novel taxon is in bold. Type sequences are labeled asterisk (*).

A total of 50 ingroup taxa from Drepanopezizaceae, Hyaloscyphaceae, Ploettnerulaceae and Vibrisseaceae were used in the phylogenetic tree analysis, of which 20 species were from the type (Fig. 1). In total, 31 isolates contained all extant species that have available molecular data within Drepanopezizaceae. The combined LSU and ITS yield a 1409 bp alignment, with the best substitution models for each summarised as TIM2e+I+G4 and TIM2+F+R3, respectively.

Phylogenetic analysis demonstrated that *Diplocarpon* divided into two phylogenetically close relative clades, *Diplocarpon* and *Entomosporium*. *Diplocarpon* clade is composed of *D.coronariae* (from China, Japan, Korea and the USA), *D.earlianum* (unknown country) and *D.rosae* (from China, Germany and an unknown country). Those three species have common characteristics of two-celled conidia. The new species *Entomosporiumdichotomanthes* (from China), along with *E.mespili* (from England, Korea and an unknown country) and *E.mespilicola* (from China) consisted of clade *Entomosporium*, which showed insect-like conidia. Moreover, *Hymenulacerealis* and *Pseudopezizamedicaginis* were within Ploettnerulaceae.

**Figure 2.**
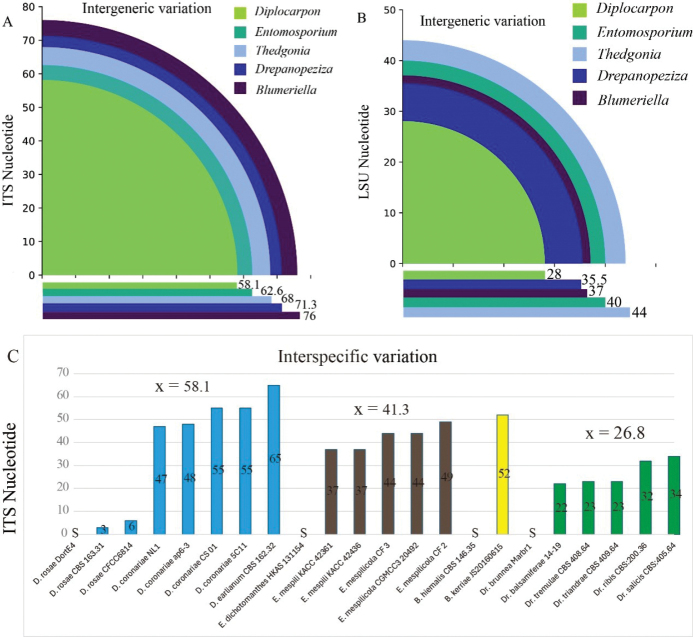
Intergeneric and interspecific variation analysis **A** mean of ITS sequence variation within genera **B** means of LSU sequence variation within genera **C**ITS sequence variation of the query sequence and the subject, “S” is the subject, “x” is the mean value of nucleotide variation within species.

Sequence comparison reveals the intergeneric and interspecific variation (Fig. 2). ITS sequence shows a high nucleotide variation within *Diplocarpon*, with an average of 58.1, compared to *Diplocarpon*, the *Entomosporium*, *Blumeriella*, *Drepanopeziza* and *Thedgonia* have an average of 62.6, 68, 71.3 and 76, respectively. The sequence comparison results align with the phylogenetic analysis, indicating that the closely related species exhibit less nucleotide variation. The LSU sequences have a lower variation. The *Diplocarpon* has an average of 28, and the *Drepanopeziza*, *Blumeriella*, *Entomosporium* and *Thedgonia* have an average of 35.5, 37, 40, and 44, respectively. The interspecific variation of *Drepanopeziza* and *Entomosporium* is 26.8 and 41.3.

### ﻿Taxonomy

#### ﻿ Drepanopezizaceae

Taxon classificationFungiHelotialesDrepanopezizaceae

Baral

9FD072A4-2569-5706-823F-A8AABB596DB5

MycoBank No: 828889

Facesoffungi Number: FoF 05864

Fig. 3

##### Type.

*Drepanopeziza* (Kleb.) Jaap 1914.

##### Description.

***Sexual morph***: Ascomata small-sized, up to 2 mm in diameter, apothecial, cupulate, margin often protruding, with or without lobes, sessile and mostly immersed. Excipulum is composed of cells of textura angularis. Paraphyses hyaline, thin-walled, aseptate or septate, apically swollen. Asci 4–8-spored, clavate or cylindrical, apex obtuse to conical, with or without apical ring. Ascospores ellipsoid to fusoid, aseptate or 1–2-septate. ***Asexual morph***: Conidiomata solitary to gregarious or confluent, mostly epiphyllous, acervulus. Conidiogenesis holoblastic. Conidia hyaline, thin-walled.

**Figure 3.**
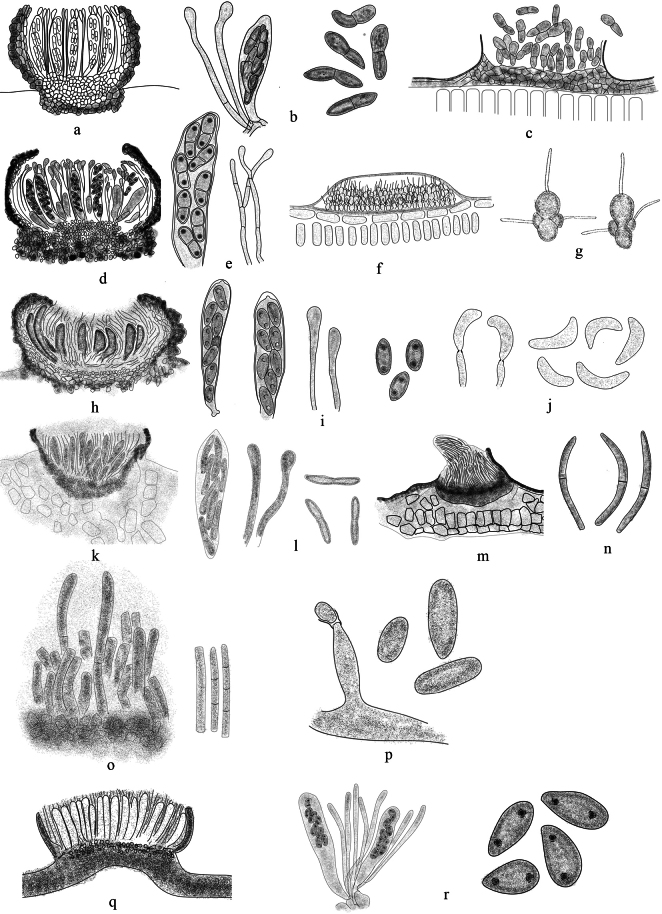
Morphology of genera in Drepanopezizaceae. *Diplocarpon*: a ascomata, b asci, paraphyses and ascospore (**a, b***D.rosae*, redraw from Wolf 1912) **c** acervulus and conidia (*D.rosae*, redraw from Lee and Shin 2000), *Entomosporium***d** ascomata **e** asci and paraphyses (**d, e***E.maculatum*, redraw from Stowell and Backus 1967) **f** acervulus **g** conidia (**f, g***E.mespilicola*, redraw from Chen et al. 2022), *Drepanopeziza***h** ascomata (*Dr.populorum*, redraw from Spiers and Hopcroft 1998) **i** asci, paraphyses and ascospore **j** conidiogenous cells and conidia (**i, j***Dr.ribis*, redraw from https://www.centrodeestudiosmicologicosasturianos.org), *Blumeriella***k** ascomata **l** asci, paraphyses and ascospore (**k, l***B.haddenii*, redraw from Williamson and Bernard 1988) **m** acervulus **n** conidia (**m, n***B.jaapii*, redraw from https://www.forestryimages.org), *Thedgonia***o** acervulus and conidia (*T.ligustrina*, redraw from Crous et al. 2009) **p***Hymenula*: conidiogenous cells and conidia (*H.gramineum* redraw from Wiese and Ravenscroft 1978), *Pseudopeziza***q** ascomata (*P.trifolii*, redraw from Kirchner and Boltshauser 1987) **r** asci, paraphyses and ascospore (*P.ribis*, redraw from https://www.pesticidy.ru/pathogens_genus/Pseudopeziza).

##### Notes.

Drepanopezizaceae was described with sexual and asexual morphs. Both life morphs were found as parasitic on leaves of various dicotyledons, and rarely on herbaceous (Johnston et al. 2019). The sexual morph is recognized by the cupulate, apothecial ascomata, and the paraphyses with swollen apical (Harada et al. 1974; Williamson and Bernard 1988; Spiers and Hopcroft 1998). The asexual morph is acervular but varies in conidial shape among genera (Crous et al. 2009; Khodadadi et al. 2022). The family name was first time used by Batista and Maia (1960), but was invalid because of unavailable diagnosis or description (Johnston et al. 2019). It was difficult to trace back the history of members accommodated in the family until Johnston et al. (2019), validated the family name based on the phylogenetic analysis (Table 2).

**Table 2.** Main versions of classification of Drepanopezizaceae and its accepted genera.

Batista and Maia (1960)	Wijayawardene et al. (2017)	Johnston et al. (2019)		Ekanayaka et al. (2019)	Wijayawardene et al. (2022)	Zhu et al. (2023)	This study
The family name was used	*Blumeriella*	Erected the family	*Blumeriella*	***Blumeriella***	*Blumeriella*	*Blumeriella*	***Blumeriella***
	*Diplocarpon*		***Diplocarpon***	***Diplocarpon***	*Diplocarpon*	***Diplocarpon***	***Diplocarpon***
	*Drepanopeziza*		*Drepanopeziza*	*Drepanopeziza*	*Drepanopeziza*	*Drepanopeziza*	***Drepanopeziza***
	*Felisbertia*		*Felisbertia*	*Felisbertia*	*Felisbertia*	*Felisbertia*	***Entomosporium***
	*Leptotrochila*		*Leptotrochila*	*Leptotrochila*	*Leptotrochila*	***Hymenula***	*Felisbertia*
	*Pseudopezicula*		*Pseudopeziza*	***Marssonina***	*Pseudopeziza*	*Leptotrochila*	*Leptotrochila*
	*Pseudopeziza*		*Spilopodia*	*Pseudopezicula*	*Spilopodia*	*Pseudopeziza*	*Spilopodia*
	*Spilopodia*		*Spilopodiella*	*Spilopodiella*	*Spilopodiella*	*Spilopodia*	*Spilopodiella*
	*Spilopodiella*		*Thedgonia*	*Spilopodia*		*Spilopodiella*	***Thedgonia***
				***Thedgonia***		*Thedgonia*	

The taxa used in the phylogenetic analysis are labeled in bold.

#### ﻿ Entomosporium

Taxon classificationFungiHelotialesDrepanopezizaceae

Lév. 1857

13299B3A-CC6E-575F-A7B8-6FC177A7FF74

MycoBank No: 8180

Facesoffungi Number: FoF 15505

##### Type.

*Entomosporiummaculatum* Lév. 1856.

##### Description.

***Sexual morph***: Ascomata small-sized, apothecial, cupulate, epiphyllous. Excipulum composed of cells of textura angularis. Paraphyses numerous, hyaline, thin-walled, septate, apically swollen, simple or branched, longer than aci. Asci 8-spored, bitunicate to uniseriate, thick-walled, clavate, short pedicel, apex obtuse, amyloid, with apical ring. Ascospores 2-celled, ellipsoidal, smooth, hyaline, thick-walled, unequal, the upper cell slightly lager. ***Asexual morph***: Conidiomata solitary to gregarious or confluent, mostly epiphyllous, acervulus. Conidiogenesis hyaline, cylindrical, holoblastic. Conidia 2–6-celled, hyaline, thin-walled, cruciform or insect-like, basal cell developed from the conidiogenous cell, cylindrical, globose to obovate, and other cells attached basal cell in both upper sides and apex, apical cell larger, globose to subglobose, lateral cells globose to ellipsoidal, smaller than the apical and basal cells, the apical and basal cells with a tubular appendage.

##### Notes.

*Entomosporium* was erected by Leveille in 1856, based on *E.maculatum* from leaves of *Pyruscommunis* (Rosaceae), and was characterized by 4-celled, cross-like conidia (Stowell and Backus 1966; Horie and Kobayashi 1980). Historically, *Entomosporium* is composed of multiple morphologically indistinguishable species. Sivanesan and Gibson combined all species to *E.mespili*, but they did not mention their taxonomic basis (Horie and Kobayashi 1980). Atkinson recorded the process by which ascospores from a cupulate fungi formed the conidia of *E.maculatum*, and named the species as *Fabraeamaculata*, while he later proposed that *F.maculata* may be identical to *E.mespili* (Atkinson 1897, 1909). Taxonomic status changed for the morphologically similar taxa, *viz. Diplocarpon*, *Entomopeziza*, *Fabraea* and *Marssonina* (Stowell and Backus 1967; Johnston et al. 2014). After versions, Jørstad (1945) combined *Diplocarpon* and *Entomosporium*, and recognized Atkinson’s collection as the type. This opinion was also discussed by Stowell and Backus (1967), but they failed the verification through experiments. The mystery of *Entomosporium* associated with sexual morph is still not confirmed by molecular data, since no new collection was found in sexual stage in recent decades.

#### ﻿ Entomosporium dichotomanthes

Taxon classificationFungiHelotialesDrepanopezizaceae

H.D. Yang, Jayaward & K.D. Hyde sp. nov.

9D8910EE-2868-5BAF-BD30-CDEC0CD06A58

Index Fungorum: IF901675

Facesoffungi Number: FoF 15506

Fig. 4

##### Etymology.

The species epithet ‘*dichotomanthes*’ refers to the host *Dichotomanthestristaniicarpa* in which the holotype was collected.

**Figure 4.**
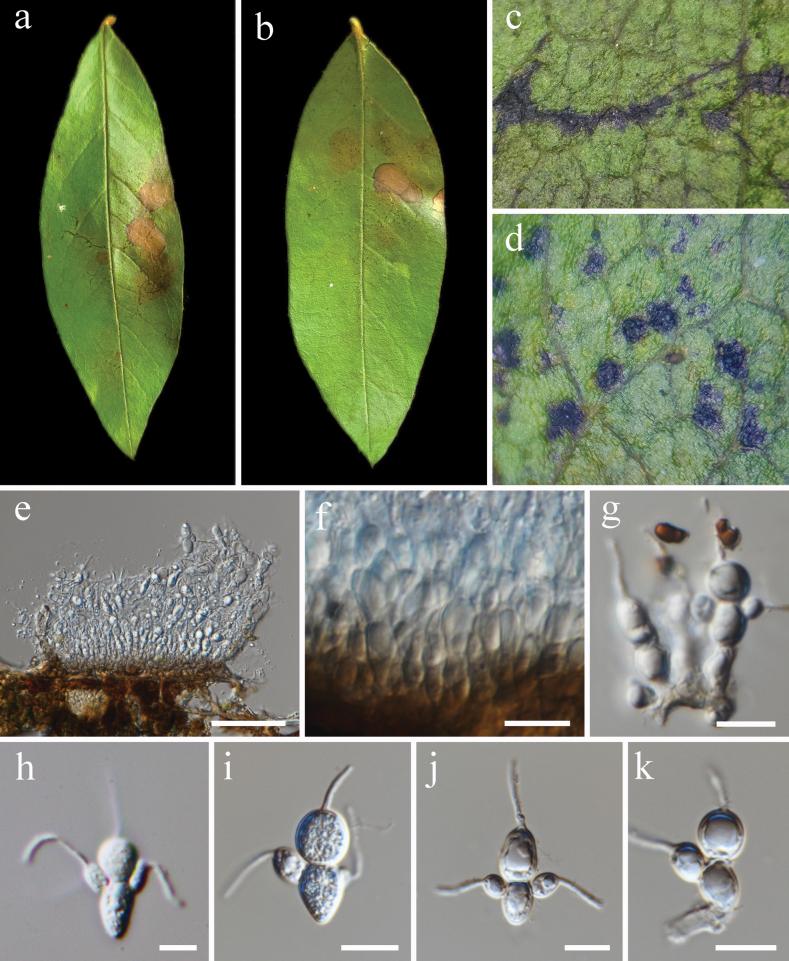
*Entomosporiumdichotomanthes* (HKAS 131154, holotype) **a, b** disease symptoms on the leaves, **c–e** conidiomata **f, g** conidiogenous cells and conidia,**h–k** conidia. Scale bars: 50 µm (**e**); 10 µm (**f–k**).

##### Holotype.

HKAS 131154.

##### Description.

Parasitic on leaf of *Dichotomanthestristaniicarpa* in terrestrial habitat. ***Leaf spots***: appear as tiny black spots or irregular black stripes on the upper side of the mature leaf when young, without injured disease symptoms. Later the spot enlarged to circular lesions or large dead areas with black edege. The area around the black spots remains green. ***Sexual morph***: Not determined. ***Asexual morph*: *Conidiomata*** dark brown to black, stromatic, acervular, epiphyllous, solitary to gregarious or confluent, subcuticular to rounded or irregular in outline, rugose, erumpent through the cuticle. ***Conidiomatal wall*** mixed with host plant tissue, of several layers loose textura angularis cell. ***Conidiophores*** hyaline to pale brown, cylindrical, branched. ***Conidiogenous cells*** 5.0–8.4 × 2.8–4.4 µm, hyaline, cylindrical, holoblastic. ***Conidia*** hyaline, 3–4-celled, cruciform, the basal cell developed from the conidiogenous cell, and other cells attached to basal cell in both upper sides and apex. Basal cells 6.7–12.1 × 4.5–8.6 (avg. = 9.9 × 7.1, n = 30) µm, cylindrical, globose to obovate. Apical cell 5.8–13.0 × 5.1–10.7 (avg. = 9.9 × 8.0, n = 30) µm, globose to subglobose, the end with a tubular appendage. Lateral cells 3.5–7.5 × 2.5–4.1 (avg. = 5.7 × 4.1, n = 20) µm, subglobose to ellipsoidal, the end with a tubular appendage.

##### Material examined.

China, Yunnan Province, Kunming City, Longchuanqiao park, 25°8'15.65"N, 102°47'13.70"E, on living leaf of *Dichotomanthestristaniicarpa*, 14 December 2021, YHD 239-5 (HKAS 131154); YHD 202.

##### Notes.

*Entomosporiumdichotomanthes* is characterized by having three to four cells of conidia. Its morphology resembles *D.mespili* and *D.mespilicola*, but has different host plants association and distribution. *E.dichotomanthes* is easily detectable on the host substrate in the mountains around the lake of Longchuanqiao Park. However, we couldn’t find this fungus on nearby plants of the host, or on other plants in the mountains. We also failed to isolate the culture by using both single spore isolation and tissue isolation methods which indicates *E.dichotomanthes* strictly rely on *D.tristaniicarpa*.

## ﻿Discussion

The taxonomic status and the phylogenetic relationship of *Diplocarpon* and *Entomosporium* in Drepanopezizaceae were assessed in this study. We included all extant species with molecular data in Drepanopezizaceae, as well as most genera of its sister family Ploettnerulaceae for the first time. Upon molecular phylogenetic analysis, *Diplocarpon* divided into two distinct clades representing *Entomosporium* and *Diplocarpon*. Sequence comparison reveals the average nucleotide variation of *Blumeriella*, *Drepanopeziza*, *Entomosporium*, and *Thedgonia* is higher than *Diplocarpon* which means intergeneric variation is greater than interspecific variation. Moreover, *Diplocarpon* and *Entomosporium* have a high nucleotide variation compared to the more speciose genus *Drepanopeziza*. Consequently, *Entomosporium* recovered separately from *Diplocarpon* and should not assign all species to *E.mespili*. On the plant host (Table 3), *D.rosae* is commonly reported on Roses, *D.earlianum* on strawberries and *D.coronaria* on apple trees. However, *Entomosporium* has a wide host range of woody plants like shrubs and trees, such as apple, hawthorn, saskatoon and pear (Holtslag et al. 2004; Nunes et al. 2016; Thurn et al. 2019). Likewise, the morphology features of conidia sustained the difference, *Entomosporium* displays insect-like conidia while *Diplocarpon* produces 2-celled conidia. Johnston et al. (2014) stated that *Entomosporium* and *Diplocarpon* are conspecific due to the linked asexual and sexual morphs, but this was not confirmed by molecular data. Conclusively, our study based on morphology coupled with molecular data supported the division of these genera. Entomosporium leaf disease is mainly associated with *Entomosporium* species (Holtslag et al. 2003; Seo et al. 2010; Nunes et al. 2016). Hence, the classification system of *Diplocarpon* was revised. We propose to recover the validity of the genus name “*Entomosporium*”, to accommodate species that have insect-like conidia species in Drepanopezizaceae. Furthermore, we introduced a new species *E.dichotomanthes* from China. Its taxonomical placement is basal in the “*Entomosporium*” clade supported by high bootstrap. The disease symptom appeared as black spots or irregular black stripes on the upper side of the mature leaf of *Dichotomanthestristaniicarpa*, which was easily recognizable. We also generated the first sequence of the *tef1-α* gene for *Entomosporium*, from *E.dichotomanthes*. However, we used only LSU and ITS sequences data in our study, since scant *tef1-α* sequences data are available for reference taxa that cannot be used in this phylogenetic analysis. The blast against NCBI shows the *tef1-α* sequences have highest similarity with *D.coronariae* (MT674914) and *Hyaloscyphafuckelii* (MT254572), gained the value of 884/948(93%) and 860/948(91%), respectively.

**Table 3.** *Diplocarpon* species documented from different countries and plant host.

Current name	Original name	Host		Disease	Symptom	Location	Reference
***D.coronaria***	*Diplocarponmali*	*Malus*	Rosaceae	Apple blotch disease	premature leaf fall of Apple	India	Goyal et al. (2018)
***D.coronaria***	*Diplocarponmali*	*Malus*	Rosaceae	Apple blotch disease	dark brown and irregularly shaped blotches or lesions	China	Lian et al. (2021)
***D.coronaria***	*Marssoninacoronaria*	*Malus*	Rosaceae	Apple blotch disease	tiny yellow spots at first, become grayish brown circular lesions	Korea	Lee et al. (2011)
***D.coronaria***	*Marssoninacoronaria*	*Malusbaccata*	Rosaceae	Leaf spot disease	initially light brown to brown lesions without a distinctive margin, later reddish black to purple, finally appearing as a yellow blotch with green islands	Korea	Lee and Shin (2000)
***D.coronaria***	*Diplocarponcoronaria*	*Malus*	Rosaceae	Apple blotch disease	dark brown and irregularly shaped blotches or lesions	Germany	Wöhner et al. (2021)
***D.coronaria***	*Diplocarponmali*	*Malus*	Rosaceae	Apple blotch disease	–	Japan	Tanaka et al. (2000)
***D.coronariae***	*Diplocarponcoronariae*	*Malus* spp.	Rosaceae	Apple blotch disease	brown to black spots, with frond-like edges or surrounded by a yellow halo	America	Khodadadi et al. (2022)
***D.earlianum***	*Diplocarponearlianum*	*Strawberry*	Rosaceae	Leaf scorch	reddish-purple lesions	Canada	Dhanvantari (1967)
***D.earlianum***	*Diplocarponearlianum*	*Strawberry*	Rosaceae	Leaf scorch disease	–	México	Garay-Serrano et al. (2021)
***D.earlianum***	*Diplocarponearlianum*	*Fragariaxananassa*	Rosaceae	Leaf scorch disease	–	America	Xue et al. (1996)
***D.fragariae***	*Diplocarponfragariae*	*Strawberry*	Rosaceae	Leaf scorch disease	–	México	Garay-Serrano et al. (2021)
***D.fragariae***	*Marssoninafragariae*	*Duchesneachrysantha*, Fragaria×ananassa, *Potentillafragarioides*, *Potentillafeyniana*	Rosaceae	Leaf spot disease	initially reddish to brown, later dark brown, central area surrounded by yellowish halo	Korea	Lee and Shin (2000)
***D.mespili***	*Diplocarponmespili*	*Pyruscommunis*	Rosaceae	Entomosporium leaf disease	reddish, purple, to dark brown spots	Southern Brazil	Nunes et al. (2016)
***D.mespili***	*Diplocarponmespili*	*Eriobotryajaponica*	Rosaceae	Entomosporium leaf disease	circular, bright red spots on young leaves, turned to purple blotches with ash brown grey centers, and coalesced to form large dead areas on leaf surfaces	Pakistan	Batool et al. (2014)
***D.mespili***	*Entomosporiummaculatum*	*Cydoniaoblonga*	Rosaceae	Entomosporium leaf disease	–	Southwestern Romania	Borcean and Imbrea (2021)
***D.mespili***	*Entomosporiummespili*	*Photiniaxfraseri*	Rosaceae	Entomosporium leaf disease	reddish-colored lesions	Georgia	Mims et al. (2000)
***D.mespili***	*Entomosporiummespili*	*Pyruscommunis*	Rosaceae	Entomosporium leaf disease	–	Southern Brazil	Bogo et al. (2018)
***D.mespili***	*Entomosporiummespili*	*Amelanchieralnifolia*	Rosaceae	Entomosporium leaf and berry spot disease	–	Canada	Holtslag et al. (2003)
***D.mespili***	*Entomosporiummespili*	*Photiniaglabra*	Rosaceae	Entomosporium leaf disease	Initially appeared as minute circular spots, later several small spots coalesced to make large necrotic blotches	Korea	Seo et al. (2010)
***D.mespili***	*Entomosporiummespili*	*Cydoniaoblonga*	Rosaceae	Entomosporium leaf disease	circular reddish-brown spots at first, coalesced producing large necrotic areas, the leaves turned yellow or reddish and fell prematurely	Southern Italy	Cariddi et al. (2009)
***D.mespili***	*Entomosporium* sp.	*Amelanchierasiatica*	Rosaceae	Entomosporium leaf disease	black shiny pustule spots	Japan	Horie and Kobayashi (1979)
***D.mespili***	*Entomosporium* sp.	*Eriobotryajaponica*	Rosaceae	Entomosporium leaf disease	yellowish or reddish spots with a greenish halo around	Japan	Horie and Kobayashi (1979)
***D.mespili***	*Entomosporium* sp.	*Pyruscommunis*	Rosaceae	Entomosporium leaf and fruit spot disease	reddish to purple at the beginning, later irregular dark brown to black necrotic patches on the leaf, sunken irregular black spot on the fruit	India	Altaf et al. (2019)
***D.mespili***	*Entomosporiummespili*	*Crataegus*	Rosaceae	small, irregularly shaped spots or larger lesions	–	America	Thurn et al. (2019)
***D.mespili***	*Diplocarponmespili*	*Pyruspyraster*	Rosaceae		–	Bulgaria	Velinova and Tashev (2017)
***D.mespilicola***	*Diplocarponmespilicola*	*Crataeguspinnatifida*	Rosaceae	Entomosporium leaf disease	brown spots	China	Chen et al. (2022)
***D.rosae***	*Diplocarponrosae*	*Rose*	Rosaceae	Black spot disease	black spots	United Kingdom	Knight and Wheeler (1977)
***D.rosae***	*Diplocarponrosae*	*Rosamultiflora*	Rosaceae	Black spot disease	black spots	Germany	Von Malek and Debener (1998)
***D.rosae***	*Diplocarponrosae*	*Rose*	Rosaceae	Black spot disease	black spots	North America	Whitaker et al. (2007)
***D.rosae***	*Diplocarponrosae*	*Rose*	Rosaceae	Black spot disease	–	Belgium	Leus et al. (2002)
***D.rosae***	*Diplocarponrosae*	*Rosarugosa*	Rosaceae	Black spot disease	black spot lesions	Canada	Bolton and Svejda (1979)
***D.saponariae***	*Diplocarponsaponariae*	*Silenelatifolia*	Caryophyllaceae	Leaf spot disease	pale yellow to pale brown spots, sometimes purple-bordered, regular or irregularly rounded, sometimes elongated	Turkey	Erdoĝdu and Hüseyin (2009)

Correspondingly, *Hymenulacerealis* and *Pseudopezizamedicaginis* were not clustered in Drepanopezizaceae in our phylogenetic tree. However, *Hymenula* was recovered within Drepanopezizaceae in Zhu et al. (2023). Further, the type material of *H.cerealis* (= *Cephalosporiumgramineum*, CBS 132.34) was used in their study and obtained a good statistical support (MLBP/BIPP = 96%/100%), in the phylogenetic analyses conducted based on the combined five-gene data set. However, only *H.cerealis* as well as a small group of taxa from both Drepanopezizaceae and Ploettnerulaceae were applied in their phylogenetic analysis. The disease caused by *Hymenula* is cephalosporium stripe on herbaceous plants that is different from Drepanopezizaceae (Wiese and Ravenscroft 1978; Zhu et al. 2023). Similar situations with *Hymenula*, *Pseudopeziza* cause black spot disease mostly found on Alfalfa and Red clover (Fabaceae), not on Rosaceae plants (Jones 1919; Meyer and Luttrell 1986; Yuan et al. 2007). The taxonomy of *Pseudopeziza* is confusing (Meyer and Luttrell 1986). There are 135 species epithets that have been linked to *Pseudopeziza* in Index Fungorum (https://www.indexfungorum.org), of which many names have been transferred to other families, such as Diaporthaceae, Ploettnerulaceae and Rhytismataceae. Only three sequences labeled as *Pseudopeziza* were accessible in the GenBank, and *P.medicaginis* (CBS 283.55) was used in this study.

Morphologically, *Hymenula* was only found in the asexual stage. Meanwhile, the asexual morph of Drepanopezizaceae does not share highly persuasive common morphological characteristics for delimiting its generic members. The morphology of *P.medicaginis* fits Drepanopezizaceae (Jones 1919), but differs in having indistinctive swollen apical paraphyses (Meyer and Luttrell 1986). Thus, we propose to exclude *H.cerealis* and *P.medicaginis* from Drepanopezizaceae and to treat them under Ploettnerulaceae.

## Supplementary Material

XML Treatment for Drepanopezizaceae

XML Treatment for Entomosporium

XML Treatment for Entomosporium dichotomanthes

## ﻿Additional information

### Author ORCIDs

Ruvishika S. Jayawardena https://orcid.org/0000-0001-7702-4885

Vinodhini Thiyagaraja https://orcid.org/0000-0002-8091-4579

Qi Zhao https://orcid.org/0000-0001-8169-0573

Kevin D. Hyde https://orcid.org/0000-0002-2191-0762

### Data availability

All of the data that support the findings of this study are available in the main text.
